# Trogocytosis by Entamoeba histolytica Mediates Acquisition and Display of Human Cell Membrane Proteins and Evasion of Lysis by Human Serum

**DOI:** 10.1128/mBio.00068-19

**Published:** 2019-04-30

**Authors:** Hannah W. Miller, Rene L. Suleiman, Katherine S. Ralston

**Affiliations:** a Department of Microbiology and Molecular Genetics, University of California, Davis, California, USA; University of California Los Angeles

**Keywords:** *Entamoeba histolytica*, trogocytosis, complement, immune evasion, amoebiasis, phagocytosis, endocytosis

## Abstract

We previously showed that Entamoeba histolytica kills human cells through a mechanism that we termed trogocytosis (“trogo-” means “nibble”), due to its resemblance to trogocytosis in other organisms. In microbial eukaryotes like E. histolytica, trogocytosis is used to kill host cells. In multicellular eukaryotes, trogocytosis is used for cell killing and cell-cell communication in a variety of contexts. Thus, nibbling is an emerging theme in cell-cell interactions both within and between species. When trogocytosis occurs between mammalian immune cells, cell membrane proteins from the nibbled cell are acquired and displayed by the recipient cell. In this study, we tested the hypothesis that through trogocytosis, amoebae acquire and display human cell membrane proteins. We demonstrate that E. histolytica acquires and displays human cell membrane proteins through trogocytosis and that this leads to protection from lysis by human serum. Protection from human serum occurs only after amoebae have undergone trogocytosis of live cells but not phagocytosis of dead cells. Likewise, mutant amoebae defective in phagocytosis, but unaltered in their capacity to perform trogocytosis, are protected from human serum. Our studies are the first to reveal that amoebae can display human cell membrane proteins and suggest that the acquisition and display of membrane proteins is a general feature of trogocytosis. These studies have major implications for interactions between E. histolytica and the immune system and also reveal a novel strategy for immune evasion by a pathogen. Since other microbial eukaryotes use trogocytosis for cell killing, our findings may apply to the pathogenesis of other infections.

## Abstract (precis)

Entamoeba histolytica causes amoebiasis, a potentially fatal diarrheal disease. Abscesses in organs such as the liver can occur when amoebae are able to breach the intestinal wall and travel through the bloodstream to other areas of the body. Therefore, understanding how E. histolytica evades immune detection is of great interest. Here, we demonstrate for the first time that E. histolytica acquires and displays human cell membrane proteins by taking “bites” of human cell material in a process named trogocytosis (“trogo-” means “nibble”), and that this allows amoebae to survive in human serum. Display of acquired proteins through trogocytosis has been previously characterized only in mammalian immune cells. Our study suggests that this is a more general feature of trogocytosis not restricted to immune cells and broadens our knowledge of eukaryotic biology. These findings also reveal a novel strategy for immune evasion by a pathogen and may apply to the pathogenesis of other infections.

## INTRODUCTION

Entamoeba histolytica is the protozoan parasite responsible for amoebiasis, a potentially fatal diarrheal disease. Amoebiasis is most prevalent in developing countries in areas with poor sanitation (1–3). A recent study found that nearly 80% of infants living in an urban slum in Bangladesh had been infected with E. histolytica by 2 years of age (4). The infection has a wide range of clinical symptoms that include asymptomatic infection, diarrhea, bloody diarrhea, and fatal abscesses outside of the intestine. Bloody diarrhea arises when amoebic trophozoites (amoebae) invade and ulcerate the intestine. Amoebae that have invaded the intestine can disseminate and cause abscesses in other tissues, most commonly in the liver. Although amoebic liver abscesses are rare, they are fatal if untreated. Little is known about the mechanisms that allow E. histolytica to evade immune detection and disseminate upon entering the bloodstream.

The parasite was named “histolytica” for its ability to damage tissue (“histo-” means “tissue”; “lytic” means “dissolving”) (5–7). Despite this name-giving property, precisely how amoebae invade and damage tissues is not clear. The most well-known virulence factor is the amoeba surface d-galactose and *N*-acetyl-d-galactosamine (Gal/GalNAc) lectin (8, 9), which mediates attachment to human cells and intestinal mucin (10–13). Surface-localized and secreted cysteine proteases contribute to proteolysis of substrates, including mucin and extracellular matrix (10–13). The profound cell killing activity of amoebae is likely to drive tissue damage. Amoebae can kill almost any type of human cell within minutes. Direct contact with human cells is required for killing to occur (8, 9). Until recently, the accepted model was that the pore-forming amoebapores act as secreted toxins (14–17). However, the contact dependence of cell killing (8, 9) and the lack of killing activity in cell lysates and supernatants (6, 7, 18) are not consistent with the presence of secreted toxins. Furthermore, transfer of amoebapores to human cells has not been demonstrated.

We previously established a new paradigm by showing that E. histolytica kills human cells through a mechanism that we termed trogocytosis (“trogo-” means “nibble”), due to its resemblance to trogocytosis in other organisms (19). During trogocytosis, amoebae kill human cells by extracting and ingesting “bites” of human cell membrane and intracellular contents (19). We defined that trogocytosis requires amoebic actin rearrangements (19). It also requires signaling initiated by the Gal/GalNAc lectin, phosphatidylinositol 3-kinase (PI3K) signaling, and an E. histolytica C2 domain-containing kinase (*Eh*C2PK) (19). By applying multiphoton imaging using explanted mouse intestinal tissue from fluorescent-membrane mice, we found that trogocytosis was required for tissue invasion, demonstrating relevance to pathogenesis (19).

Trogocytosis is not unique to E. histolytica, as it can be observed in other eukaryotes (20). Examples in microbes include reports of trogocytosis by Naegleria fowleri (21) and *Dictyostelium caveatum* (22). In multicellular eukaryotes, trogocytosis is used for a variety of cell-cell interactions in the immune system (23, 24), in the central nervous system (25, 26), and during development (27). It is not yet clear how trogocytosis can paradoxically be both a benign form of cell-cell interaction and a mechanism for cell killing. The previous paradigm was that microbes engage trogocytosis for cell killing, and trogocytosis in multicellular organisms was believed to be a benign form of cell-cell interaction. However, recent reports have now shown that neutrophils can use trogocytosis to kill parasites (28) and that neutrophils and macrophages can use trogocytosis to kill cancer cells in a form of antibody-dependent cell-mediated cytotoxicity (29, 30). Trogocytosis is therefore likely to be a conserved, fundamental form of eukaryotic cell-cell interaction that can be cytotoxic or benign, depending on the context.

One intriguing outcome of trogocytosis between mammalian immune cells is that it changes the makeup of cell surface proteins on both the donor and the recipient cell. The nibbling cell displays the acquired membrane proteins from the nibbled cell on its own surface (24, 31). Acquired membrane proteins appear as foci or patches on the recipient cell. This allows the recipient cell to take on new properties that impact its subsequent interactions with other cells (24, 31). For instance, uninfected dendritic cells can acquire and display preloaded major histocompatibility complex class II (MHC II) molecules by nibbling infected dendritic cells, and thus they can present peptides from microbes that they have not directly encountered, which has been termed “cross-dressing” (24). Transferred molecules are not limited to MHC complexes, as induced regulatory T cells can acquire cluster of differentiation (CD) molecules from mature dendritic cells, including CD80 and CD86 cells (32). It has also been shown that monocytes, NK cells, and granulocytes can acquire CD22, CD19, CD21, and CD79b from antibody-opsonized B cells (33). In addition to allowing the nibbling cell to display newly acquired membrane proteins, since membrane fragments are removed from the nibbled cell, trogocytosis also affects the nibbled cell by effectively downregulating surface proteins (34).

Since mammalian immune cells acquire and display membrane proteins through trogocytosis, we hypothesized that amoebae may acquire and display human cell membrane proteins. Amoebic display of human proteins would have significant implications for host-pathogen interactions. We predicted that one outcome of amoebic human cell protein display might be the inhibition of lysis by human complement. Previous studies have suggested that amoebae become more resistant to complement after interacting with host cells or tissues and that complement resistance appears to involve proteins on the amoeba surface (35–37).

Here, we show that E. histolytica acquires and displays human cell membrane proteins. Acquisition and display of human cell membrane proteins requires actin and direct contact and is associated with subsequent protection from lysis by human serum. Protection from human serum occurs after amoebae have undergone trogocytosis, but not phagocytosis, suggesting that protection is not generally associated with ingestion. Collectively, these findings support a new model in which amoebae acquire and display human cell membrane proteins through trogocytosis, leading to protection from lysis by human serum complement. These studies have major implications for interactions between E. histolytica and the immune system.

(This article was submitted to an online preprint archive [38].)

## RESULTS

### Amoebae acquire and display human cell membrane proteins.

**FIG 1:**
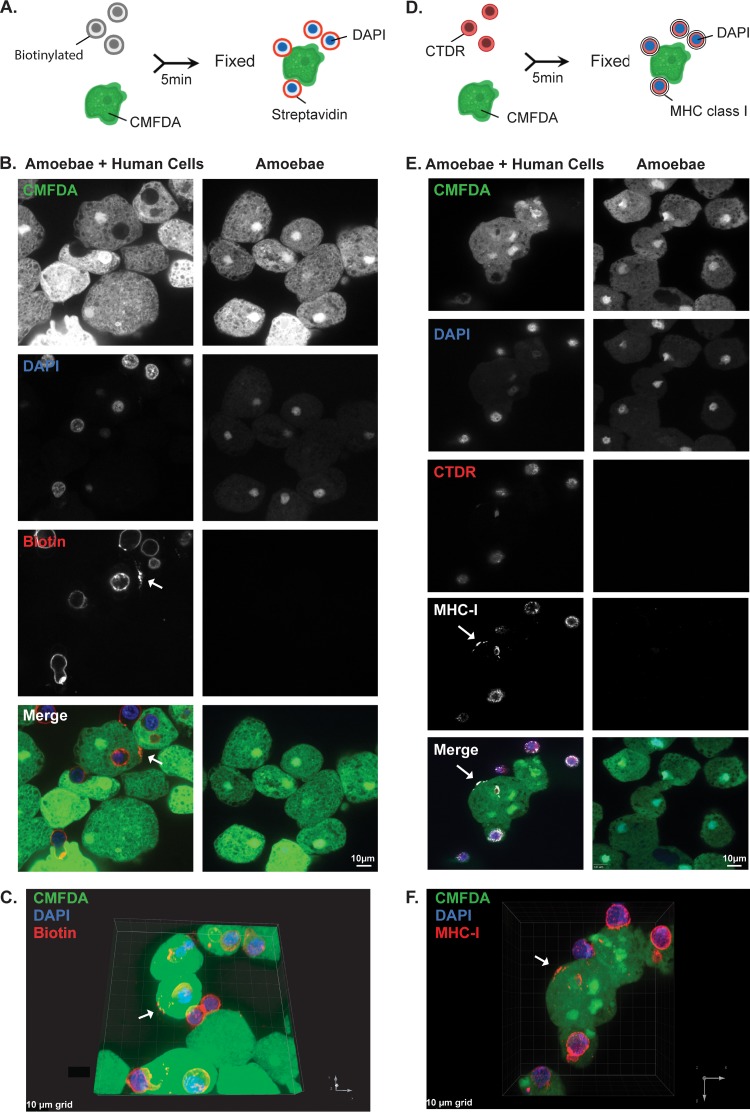
Following interaction with human cells, human cell membrane proteins are displayed by amoebae. (A) Human cell membrane proteins were labeled with biotin prior to coincubation with CMFDA-labeled amoebae. Cells were coincubated for 5 min and immediately fixed. Following fixation, samples were labeled with fluorescently conjugated streptavidin and DAPI. (B) Representative images of amoebae incubated alone or coincubated with biotinylated human cells. Amoebae are shown in green, and streptavidin is shown in red. Nuclei are shown in blue. Arrow indicates a patch of biotin-streptavidin localized to the amoeba surface. (C) Three-dimensional rendering of Z stack images taken from panel B. The arrow indicates transferred biotin. (D) Human cells were labeled with CellTracker deep red (CTDR) prior to coincubation with CMFDA-labeled amoebae. Cells were coincubated for 5 min and immediately fixed. Following fixation, samples were labeled with DAPI and MHC I molecules were detected using immunofluorescence. (E) Representative images of amoebae incubated alone or coincubated with CTDR-labeled human cells. Amoebae are shown in green, human cell cytoplasm is shown in red, MHC I molecules are shown in white, and nuclei are shown in blue. The arrow indicates a patch of MHC I molecules localized to the amoeba surface. (F) Three-dimensional rendering of Z stack images taken from panel E. The arrow indicates transferred MHC I. For panels B to F, images were collected from 4 independent experiments. For biotin experiments, 76 images of amoebae with human cells and 21 images of amoebae alone were collected. For MHC I experiments, 83 images of amoebae with human cells and 40 images of amoebae alone were collected.

We first asked whether trogocytosis by E. histolytica could result in transfer of human cell membrane proteins to the cell membrane of an amoeba. Human Jurkat T cells were surface biotinylated and then coincubated with amoebae. After coincubation, cells were fixed and labeled with fluorescently conjugated streptavidin (Fig. 1A). Since cells were not permeabilized, this approach required human cell proteins to be surface exposed and to retain correct orientation for recognition by streptavidin. After 5 min of coincubation, patches of streptavidin-labeled human cell proteins were detected on the surfaces of amoebae (Fig. 1B and C, arrows). As with immune cell “cross-dressing” (24), the biotin-streptavidin label appeared as foci on the amoeba surface. To track an individual human cell membrane protein, immunofluorescence was used to detect human major histocompatibility complex class I (MHC I) molecules (Fig. 1D). Following coincubation, cells were fixed without permeabilization, and MHC I molecules were detected using a monoclonal antibody. Similar to the biotin-streptavidin labeling experiments, MHC I molecules were detected in foci on the surfaces of amoebae after 5 min of coincubation (Fig. 1E and F). Thus, human cell membrane proteins were acquired and displayed by amoebae.

### Acquisition and display of human cell membrane proteins require actin.

Trogocytosis by E. histolytica requires actin rearrangements and is inhibited by cytochalasin D (19). Therefore, we asked whether acquisition of human cell membrane proteins required actin. Imaging flow cytometry was used to quantify biotinylated human cell membrane proteins on the amoeba surface. It was important to distinguish between amoebae that displayed human cell membrane proteins and amoebae that were attached to intact, extracellular human cells. While the latter amoebae might also display human cell membrane proteins, we focused our analysis on images that lacked extracellular human cells, as this allowed for the highest stringency in quantifying displayed human cell membrane proteins. Since human cell nuclei are not internalized by amoebae during trogocytosis (19), human cell nuclei were fluorescently labeled, and this was used to gate images that contained or lacked extracellular human cells.

**FIG 2:**
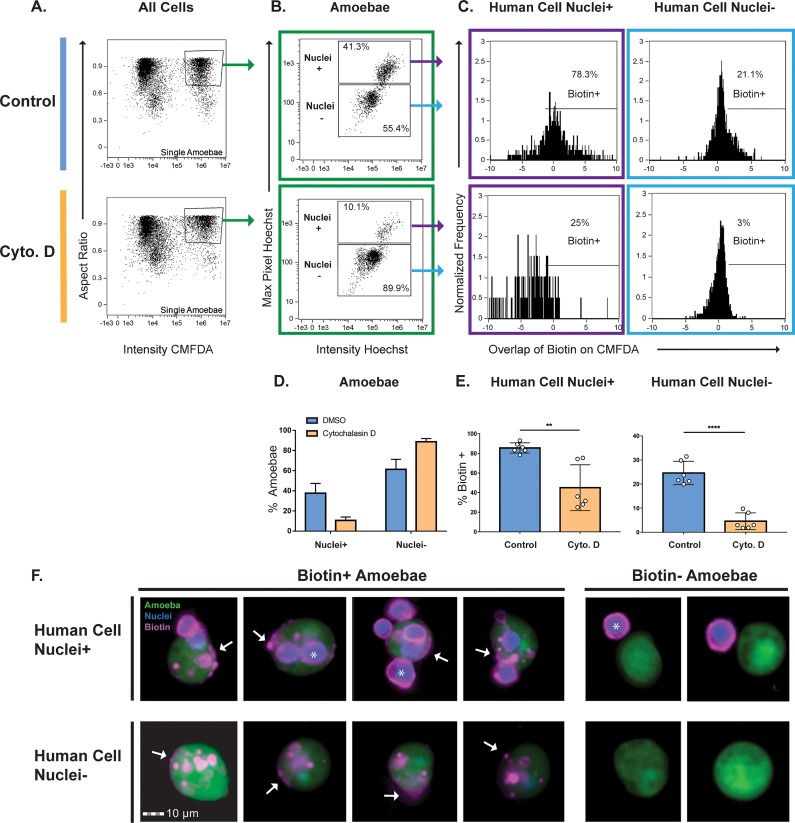
Acquisition of human cell membrane proteins is inhibited with cytochalasin D treatment. CMFDA-labeled amoebae were pretreated with either cytochalasin D (Cyto. D) or DMSO (Control) and were then combined with Hoechst labeled human cells. Immediately after coincubation, cells were placed on ice to halt ingestion and stained with fluorescently conjugated streptavidin. Samples were quantitatively analyzed using imaging flow cytometry, with 10,000 images collected for each sample. (A) Gate used to identify single amoebae from total cells. Focused cells were gated on single amoebae using the aspect ratio and intensity of CMFDA fluorescence. (B) Representative plots of images with and without human cell nuclei (high- and low-Hoechst populations) are shown. The high-Hoechst population contained images of amoebae with human cells, and the low-Hoechst population contained images of amoebae without human cells. (C) The overlap of biotin and CMFDA fluorescence was measured, and biotin-positive images were gated. Representative plots of DMSO- and cytochalasin D-treated samples are shown. (D) Quantification of plots from panel B. DMSO-treated samples are shown in blue, and cytochalasin D-treated samples are shown in orange. (E) Quantification of plots from panel C. (F) Representative images of the populations shown in panel C. Amoebae are shown in green, cell nuclei are shown in blue, and biotin is shown in magenta. Arrows indicate patches of transferred biotin. Whole human cells with stained nuclei are marked with asterisks. Six replicates across 3 independent experiments were performed.

Human cell nuclei were labeled with Hoechst dye, and human cell membrane proteins were biotinylated prior to coincubation with amoebae. After gating of single amoebae out of total cells (Fig. 2A; see also Fig. S1 in the supplemental material), Hoechst staining was used to gate images of amoebae with and without extracellular human cells (Fig. 2B, D, and F). Next, the extent of overlap of fluorescent streptavidin and individual amoebae was quantified (Fig. 2C and E). In the dimethyl sulfoxide (DMSO)-treated control amoebae, 25% of amoebae contained foci of biotin labeling, while in the cytochalasin D-treated amoebae, 5% of amoebae contained foci of biotin labeling (Fig. 2E). Thus, amoebae acquire and display human cell membrane proteins through an actin-dependent process, consistent with trogocytosis. Moreover, only 3% of biotin-positive amoebae had undergone phagocytosis (Fig. S2), consistent with a predominant role for trogocytosis in the acquisition and display of human cell membrane proteins.

10.1128/mBio.00068-19.1 FIG S1 Gating strategy used to quantify transferred biotin. Gating strategy used to quantify biotin-positive amoebae. Focused cells were gated from all collected events. Next, single cells were gated, and then single amoebae were gated. Single amoebae were divided into high-Hoechst and low-Hoechst populations to identify images with and without human cell nuclei. Finally, biotin-positive amoebae were gated from images with and without human cell nuclei. Download FIG S1, TIF file, 1.3 MB. Copyright © 2019 Miller et al. 2019 Miller et al. This content is distributed under the terms of the Creative Commons Attribution 4.0 International license.

10.1128/mBio.00068-19.2 FIG S2 Analysis used to quantify the background level of phagocytosis in trogocytosis assays. The imaging flow cytometry data from the experiments shown in Fig. 2 were used to quantify the level of phagocytosis in trogocytosis assays. CMFDA-labeled control DMSO-treated amoebae were combined with Hoechst-labeled human cells. Immediately after coincubation, cells were placed on ice to halt ingestion and stained with fluorescently conjugated streptavidin. Samples were quantitatively analyzed using imaging flow cytometry, with 10,000 images collected for each sample. (A) Single amoebae were gated from the total number of cells. Next, biotin-positive amoebae were gated. (B) Human cell nuclei (asterisks) that were surrounded by a biotin/streptavidin ring (arrow) were considered extracellular, while human cell nuclei that lacked a biotin ring were considered internalized. Thus, amoebae that were associated with extracellular human cells were considered phagocytosis negative, while amoebae associated with internalized human cells were considered phagocytosis positive. Amoebae that were biotin positive (arrowhead) without associated human cell nuclei were considered phagocytosis negative. Some amoebae were out of focus or were associated with too many human cells to be reliably scored; thus, these images were left unscored. Representative images of phagocytosis-positive, phagocytosis-negative, and unscored amoebae are shown. (C) Among three independent experiments, the average level of phagocytosis was 3% (range of 2 to 5%). (D) Table showing the raw data for the analysis in panel C. One hundred images each from three separate experiments (300 total scored images, plus unscored images as indicated) were counted. Images were counted independently by two different researchers, and the counts were averaged. Download FIG S2, TIF file, 2.0 MB. Copyright © 2019 Miller et al. 2019 Miller et al. This content is distributed under the terms of the Creative Commons Attribution 4.0 International license.

### Interaction with human cells leads to protection from lysis by human serum.

**FIG 3:**
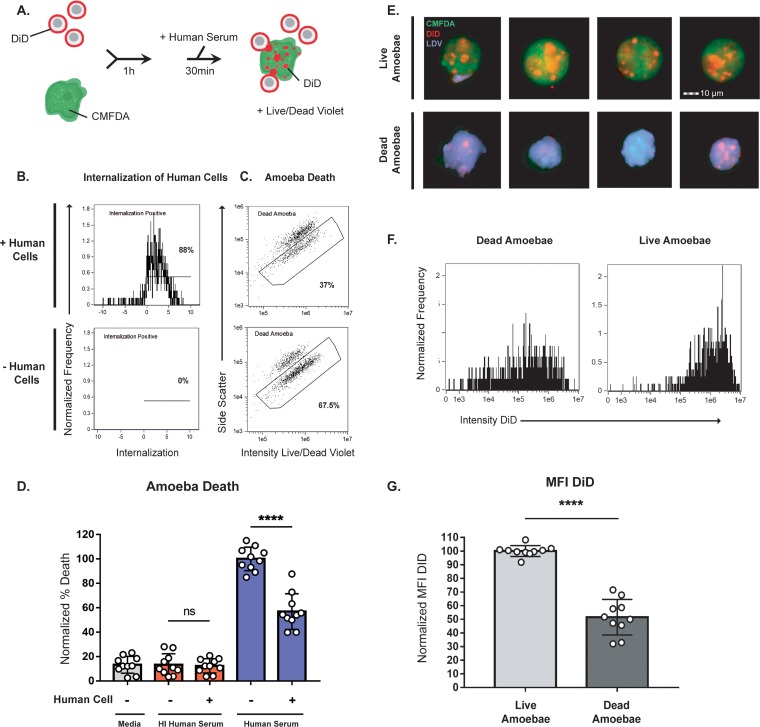
Interaction with human cells leads to protection from lysis by human serum. (A) CMFDA-labeled amoebae were incubated alone or in the presence of DiD-labeled human cells for 1 h. Cells were then exposed to either active human serum, heat-inactivated human serum, or M199s medium. Following exposure to serum, samples were stained with Live/Dead violet, and viability was quantified using imaging flow cytometry, with 10,000 images collected for each sample. (B) Representative plots showing internalization of human cells from amoebae incubated with human cells or in the absence of human cells. (C) Representative plots comparing amoebic death from the conditions shown in panel B. (D) Quantification of amoebic death for all experimental conditions. Cells exposed to M199s medium are shown in gray, to heat-inactivated (HI) human serum in red, and to active human serum in blue. Percentages of dead amoebae were normalized to numbers of dead amoebae in the amoeba-alone samples that were treated with active human serum. (E) Representative images of live and dead amoebae from amoebae coincubated with human cells and exposed to active human serum. Amoebae are shown in green, human cell membranes in red, and dead cells in violet. (F) Representative histograms showing the mean fluorescence intensity (MFI) of DiD in live and dead amoebae from samples exposed to human serum. (G) Quantification of the DID MFI shown in panel F. Ten replicates across 5 independent experiments were performed. ns, not significant.

The acquisition and display of human cell membrane proteins has many potential implications for host-parasite interactions. One possible implication is in resistance to lysis by complement in human serum, particularly since it has been previously suggested that ingestion of human erythrocytes protects amoebae from lysis by human complement (35). Amoebae preferentially perform trogocytosis on live human cells (19); therefore, amoebae were incubated in the presence or absence of live human cells and then exposed to human serum (Fig. 3A; Fig. S3). Using imaging flow cytometry, amoeba viability (Fig. 3C and D) and trogocytosis were simultaneously measured (Fig. 3B; Fig. S4). Amoebae that had interacted with live human cells and had thus undergone trogocytosis and acquired human cell membrane proteins were quantitatively protected from lysis by human serum (Fig. 3C and D; Fig. S5). Among amoebae that had been incubated with human cells, amoebae that were lysed by human serum had undergone quantitatively less trogocytosis than amoebae that survived exposure to human serum (Fig. 3E to G). Therefore, trogocytosis is associated with subsequent protection from lysis by human serum.

10.1128/mBio.00068-19.3 FIG S3 Optimization of complement assay. The ability of unsupplemented human serum from different vendors to lyse amoebae was tested at various concentrations for 30 min, 1 h, and 2 h at 35°C. Samples were labeled with the viability dye Live/Dead violet and percentages of dead amoebae were determined using imaging flow cytometry. The percentage of dead amoebae was not normalized. (A) Sigma male AB serum. Note that serum was stored at −20°C instead of −80°C. (B) Sigma complement serum human lyophilized powder. (C) Innovative Research pooled normal human complement serum. (D) Valley Biomedical human complement (serum). (E, F) The lysis of increasing concentrations of serum from Innovative Research and Valley Biomedical was tested with the addition of 150 µM CaCl_2_ and 150 µM MgCl_2_ for 1 h at 35°C. Download FIG S3, TIF file, 2.0 MB. Copyright © 2019 Miller et al. 2019 Miller et al. This content is distributed under the terms of the Creative Commons Attribution 4.0 International license.

10.1128/mBio.00068-19.4 FIG S4 Gating strategy used in the serum lysis assay. Focused cells were gated from all collected events. Next, focused events were divided into gates that contained either debris and human cells or single amoebae. Single amoebae positive for human cells were gated, and then internalization of human cells was measured. The percentage of dead amoebae was gated from single amoebae. Download FIG S4, TIF file, 2.1 MB. Copyright © 2019 Miller et al. 2019 Miller et al. This content is distributed under the terms of the Creative Commons Attribution 4.0 International license.

10.1128/mBio.00068-19.5 FIG S5 Nonnormalized data from the serum lysis assay shown in Fig. 3. (A and B) Amoebic lysis was varied and fell into two groups, low lysis (A) and high lysis (B). (C) Lysis from all nonnormalized data. (D) Lysis from all data normalized to the condition with amoebae incubated in the absence of human cells and with exposure to active human serum. Download FIG S5, TIF file, 1.2 MB. Copyright © 2019 Miller et al. 2019 Miller et al. This content is distributed under the terms of the Creative Commons Attribution 4.0 International license.

10.1128/mBio.00068-19.6 FIG S6 Centrifugation does not rescue the defect in cytochalasin D-treated amoebae. The experiments shown in Fig. 5 were repeated with the addition of a centrifugation step to force contact between amoebae and human cells at the start of the coincubation. CMFDA-labeled amoebae and DiD-labeled human cells were centrifuged together at 400 × *g* for 8 min and then coincubated for 1 h, or amoebae were centrifuged and incubated in the absence of human cells as a control. Samples were then exposed to active human serum for 1 h, stained with Live/Dead violet viability dye, and quantitatively analyzed using imaging flow cytometry. Ten thousand images were collected for each sample. (A) Amoebae were either pretreated with cytochalasin D (dark gray) or DMSO (light gray) for 1 h. The internalization of human cells was quantified. (B) The quantification of amoebic death is shown. The percentages of dead amoebae were normalized to the number of dead amoebae in DMSO-treated, amoeba-alone samples. Eight replicates across 4 independent experiments were performed. Download FIG S6, TIF file, 0.9 MB. Copyright © 2019 Miller et al. 2019 Miller et al. This content is distributed under the terms of the Creative Commons Attribution 4.0 International license.

### Protection from human serum lysis is dependent on contact with human cells.

**FIG 4:**
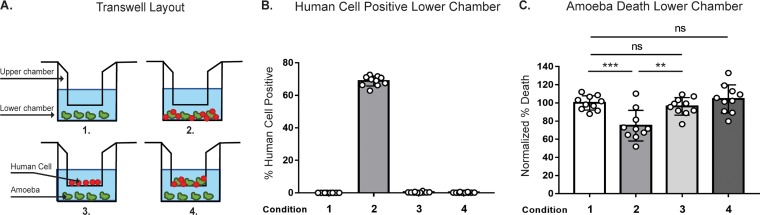
Protection from human serum lysis is dependent on contact with human cells. (A) Depiction of each transwell condition used in panels B to C. CMFDA-labeled amoebae and DiD-labeled human cells were incubated alone, together, or separately under four different transwell conditions. Condition 1, amoebae alone in the lower chamber; condition 2, amoebae and human cells together in the lower chamber; condition 3, human cells in the upper chamber and amoebae in the lower chamber; and condition 4, amoebae and human cells together in the upper chamber and amoebae in the lower chamber. Cells were coincubated in transwells for 1 h, and then cells from the lower chambers were harvested, exposed to human serum, and analyzed. Viability was assessed using Live/Dead violet dye and imaging flow cytometry (B) Quantification of human-cell-positive amoebae under conditions 1 to 4 from panel A. (C) Quantification of amoebic death under conditions 1 to 4. Percentages of dead amoebae were normalized to numbers of dead amoebae under condition 1 (amoebae alone). Ten replicates across 5 independent experiments were performed.

We next asked if protection from serum lysis required direct contact between amoebae and human cells in order to determine if protection is a consequence of trogocytosis or if protection could be acquired through secreted human cell proteins or exosomes. Amoebae and human cells were coincubated in transwell dishes, with or without direct contact (Fig. 4A). Human cells were not able to pass through transwell membranes (Fig. 4B). Protection from complement lysis occurred only when amoebae and human cells were incubated together in the same chamber of the transwell, not when they were separated (Fig. 4C). Protection from human serum thus required direct contact between amoebae and human cells, supporting a requirement for trogocytosis in the acquisition of protection.

### Protection from human serum requires actin.

**FIG 5:**
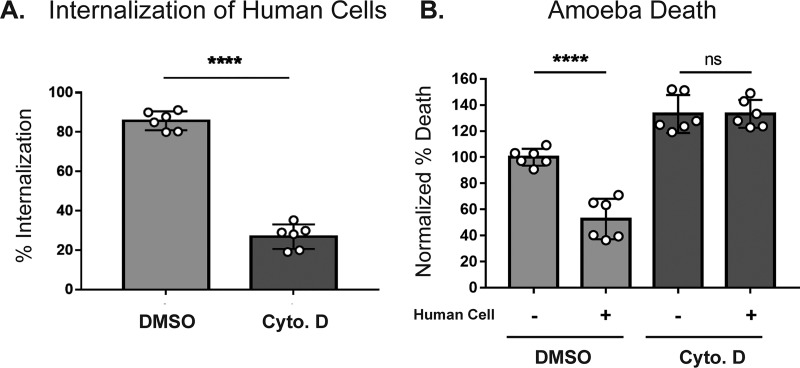
Protection from human serum is actin dependent. CMFDA-labeled amoebae were incubated alone or in the presence of DiD-labeled human cells for 1 h and then exposed to active human serum. Samples were then stained with Live/Dead violet viability dye and analyzed by imaging flow cytometry. (A) Amoebae were either pretreated with cytochalasin D (dark gray) or DMSO (light gray) for 1 h. The internalization of human cells was quantified. (B) The quantification of amoebic death is shown. Percentages of dead amoebae were normalized to the numbers of dead amoebae in the amoeba-alone DMSO-treated samples. Six replicates across 3 independent experiments were performed.

Since acquisition and display of human cell membrane proteins requires actin (Fig. 2), we next asked if treatment with cytochalasin D would also abrogate protection from human serum. Amoebae were treated with cytochalasin D, incubated in the presence or absence of human cells, and then exposed to human serum. Imaging flow cytometry was used to simultaneously measure trogocytosis (Fig. 5A) and amoeba viability (Fig. 5B). Amoebae that were treated with cytochalasin D were impaired in their ability to undergo trogocytosis and were not protected from serum lysis after coincubation with human cells. Since cytochalasin D inhibits cell motility, centrifugation was used to bring amoebae and human cells into contact (Fig. S6). Under these conditions, cytochalasin D-treated amoebae were still impaired in their ability to undergo trogocytosis and were not protected from subsequent serum lysis. Thus, centrifugation does not rescue the defect in cytochalasin D-treated amoebae. Actin rearrangements are therefore required for subsequent protection from lysis by human serum.

### Protection occurs after trogocytosis and does not occur after phagocytosis.

**FIG 6:**
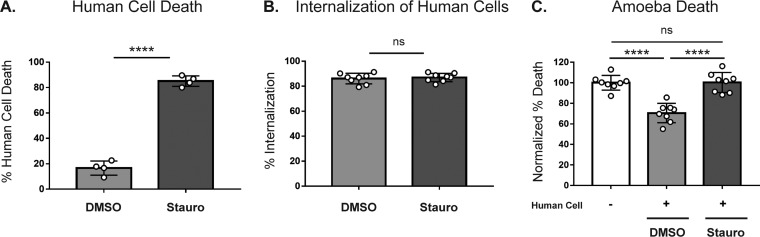
Protection is associated with trogocytosis but not phagocytosis of human cells. (A) Human cells were pretreated with staurosporine (Stauro; dark gray) or DMSO (light gray). The human cell viability before coincubation is shown. (B) Quantification of human cell internalization by amoebae. (C) Quantification of amoebic death. Percentages of dead amoebae were normalized to the numbers of dead amoebae in the amoeba-alone samples. Eight replicates across 4 independent experiments were performed.

To ask if protection from human serum specifically occurs after trogocytosis or if any form of ingestion leads to protection from serum, we compared amoebae that had undergone trogocytosis with those that had undergone phagocytosis. We previously showed that amoebae undergo trogocytosis of live human cells and, in contrast, undergo phagocytosis of prekilled human cells (19). Therefore, we asked if phagocytosis of prekilled cells could also provide protection from complement lysis. Human cells were prekilled by pretreating them with staurosporine to induce apoptosis (Fig. 6A). Amoebae were coincubated with live or prekilled human cells or incubated in the absence of human cells. Amoebae that had undergone trogocytosis or phagocytosis ingested similar amounts of human cell material (Fig. 6B); however, amoebae were protected from lysis only by human serum after undergoing trogocytosis (Fig. 6C). Therefore, protection from lysis by human serum occurs specifically after trogocytosis of live cells.

**FIG 7:**
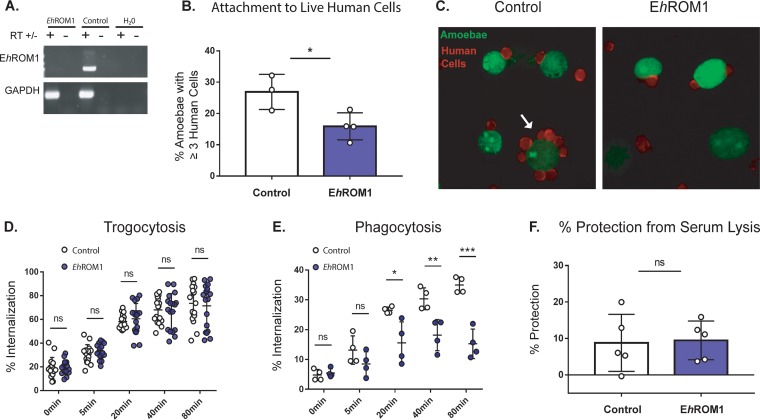
*Eh*ROM1 knockdown mutants defective in phagocytosis but not trogocytosis are protected from serum lysis. Amoebae were stably transfected with an *Eh*ROM1 knockdown plasmid (*Eh*Rom1) or a vector control plasmid (Control). (A) Silencing of *Eh*ROM1 was verified by using reverse transcriptase (RT) PCR. RT was included (+) or omitted (–) as a control. GAPDH (glyceraldehyde-3-phosphate dehydrogenase) was used to control for loading. (B) *Eh*ROM1 and vector control transfectants were incubated on ice with live human cells for 1 h and then fixed and analyzed using confocal microscopy. The percentage of amoebae with 3 or more attached human cells for each condition is displayed; a vector control is shown with open bars, and the *Eh*ROM1 knockdown mutant is shown with blue bars. Four replicates across 2 independent experiments were performed. Twenty images were collected per slide, and 195 to 252 individual amoebae were counted per condition. (C) Representative images from panel B. Amoebae are shown in green, and human cells are shown in red. The arrow indicates an amoeba with a rosette of attached human cells. (D) CMFDA-labeled *Eh*ROM1 knockdown mutants (blue circles) or vector control transfectants (open circles) were incubated alone or in the presence of live DiD-labeled human cells for 0, 5, 20, 40, or 80 min. Internalization of human cell material was quantified using imaging flow cytometry. Twenty replicates across 10 independent experiments were performed. (E) CMFDA-labeled *Eh*ROM1 knockdown mutants (blue circles) or vector control transfectants (open circles) were incubated alone or in the presence of heat-killed CTDR-labeled human cells for 0, 5, 20, 40, or 80 min. Internalization of human cell material was quantified using imaging flow cytometry. Four replicates across 2 independent experiments were performed. (F) *Eh*ROM1 (blue bar) or vector control (open bar) amoebae were coincubated with live human cells for 1 h and then exposed to human serum. Viability was assessed using Live/Dead violet dye and imaging flow cytometry. Percent protection was calculated by subtracting the total lysis of amoebae coincubated with human cells from the total lysis of amoebae incubated alone. Nine to 10 replicates across 5 independent experiments were performed. Protection data are means of results from 2 replicates per experiment from all 5 experiments.

To further distinguish between requirements for trogocytosis and phagocytosis, we tested mutants deficient in E. histolytica rhomboid protease 1 (*Eh*ROM1) (EHI_197460), a protease with roles in attachment and ingestion (39, 40). *Eh*ROM1 mutants have been shown to be deficient in phagocytosis, pinocytosis, and attachment to live cells (39, 40). Furthermore, silencing of *Eh*ROM1 does not change susceptibility to serum lysis, making these mutants an ideal tool for testing the effects conferred by ingestion of human cells (39, 40). We generated stable *Eh*ROM1 knockdown mutants (Fig. 7A), which were deficient in attachment to healthy human cells (Fig. 7B and C), consistent with the results of previous studies (40). Also consistently with previous studies, *Eh*ROM1 mutant amoebae incubated alone were not more susceptible to serum lysis than control amoebae (Fig. S7B). *Eh*ROM1 mutants did not exhibit a trogocytosis defect (Fig. 7D; Fig. S8). As expected, *Eh*ROM1 mutants were defective in phagocytosis (Fig. 7E). After trogocytosis, *Eh*ROM1 mutants were no more or less protected from lysis by human serum than control amoebae (Fig. 7F; Fig. S7). Therefore, a mutant deficient in phagocytosis does not exhibit a difference in protection from serum, further supporting the idea that phagocytosis is not involved in resistance to lysis by human serum. Moreover, resistance to lysis by human serum is not associated with simple attachment to human cells, since *Eh*ROM1 mutants are impaired in binding to live human cells but still exhibit no difference in resistance to human serum. Together, these findings further underscore the finding that protection from lysis by human serum is not associated with phagocytosis.

10.1128/mBio.00068-19.7 FIG S7 Internalization of human cells and amoebic death, as well as additional data, from the serum lysis assay shown in Fig. 7. (A) Internalization of human cells by vector control transfectants (white bar) or *Eh*ROM1 knockdown mutants (purple bar); (B) percentages of normalized amoeba death under the conditions where amoebae were incubated alone; (C) nonnormalized amoebic death from all conditions. Download FIG S7, TIF file, 0.6 MB. Copyright © 2019 Miller et al. 2019 Miller et al. This content is distributed under the terms of the Creative Commons Attribution 4.0 International license.

10.1128/mBio.00068-19.8 FIG S8 (A) Gating strategy used in the trogocytosis and phagocytosis assays shown in Fig. 7. Shown are sample data from a trogocytosis assay, where CMFDA-labeled amoebae were incubated with live DiD-labeled human cells. For phagocytosis assays, CMFDA-labeled amoebae were incubated with CTDR-labeled heat-killed human cells. Focused cells were gated from all collected events. Next, single cells were gated, and then single amoebae were gated. Amoebae positive for human cells were gated, and internalization of human cells was measured. (B) Sample data from a trogocytosis assay, with representative plots from the vector control condition showing internalization of human cells over time as well as representative images collected at each time point. Download FIG S8, TIF file, 1.3 MB. Copyright © 2019 Miller et al. 2019 Miller et al. This content is distributed under the terms of the Creative Commons Attribution 4.0 International license.

**FIG 8:**
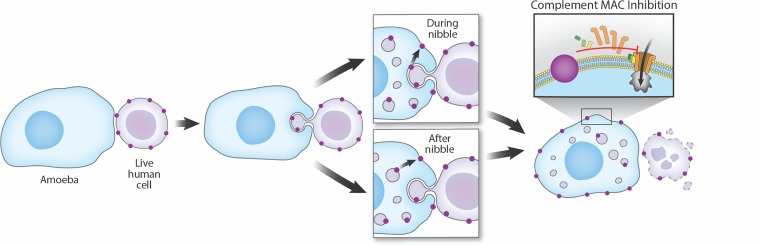
Proposed model of protection from serum lysis. Amoebae encounter live human cells while invading the intestine or disseminating in the bloodstream and perform trogocytosis. Trogocytosis leads to acquisition and display of human cell membrane proteins on the amoeba surface. One potential mechanism for the acquisition and display of human cell membrane proteins is through fusion of the amoebic and human cell plasma membranes during trogocytosis (during nibble). Human cell proteins might be directly transferred to the amoeba surface through membrane fusion at the site of trogocytosis without being first internalized. Another potential mechanism is through internalization of bites during trogocytosis (after nibble). The ingested membrane proteins might then be trafficked to the amoeba surface. Display of human cell membrane proteins then protects the amoebae from lysis in the blood by inhibiting the complement cascade.

Collectively, these results support a new model of immune evasion in which amoebae perform trogocytosis on live human cells and through trogocytosis, acquire and display human cell membrane proteins. Display of human cell membrane proteins then leads to protection from human serum, most likely by inhibiting complement-mediated lysis (Fig. 8).

## DISCUSSION

Our studies revealed that amoebae acquire and display human cell membrane proteins. This process is actin dependent and is associated with resistance to lysis by human serum. Protection from lysis by human serum requires direct contact between amoebae and human cells, is actin dependent, and is specifically associated with trogocytosis, not phagocytosis. Collectively, these data suggest that amoebae acquire and display human cell membrane proteins through trogocytosis and that this leads to protection from lysis by human serum complement.

Complement resistance by amoebae is relevant to invasive disease. Once amoebae have invaded intestinal tissue, they can spread from the intestine to the liver through the portal vein (41), and they can ingest erythrocytes (42); thus, they are capable of surviving in the bloodstream. A study that depleted complement by using cobra venom factor in the hamster model of amoebic liver abscess found that loss of complement was correlated with greater severity of liver lesions (43). Additionally, serum from women was more effective in killing amoebae then serum from men, and men are known to be more susceptible to invasive amoebiasis (44). Furthermore, pathogenic amoebae have been shown to resist complement. E. histolytica appears to evade complement deposition, while the closely related nonpathogenic species *Entamoeba dispar* does not (45). Similarly, amoebae isolated from patients with invasive infection resist complement, while strains isolated from asymptomatic patients are complement sensitive (46).

Previous studies have hinted that amoebae become more resistant to complement after interacting with host cells or tissues and that complement resistance involves proteins on the amoeba surface. It has previously been demonstrated that amoebae that were made resistant to complement lysis by hamster liver passage lost resistance after treatment with trypsin (36), suggesting that complement resistance is associated with proteins on the amoeba surface. It has also been shown that amoebae acquire serum resistance after ingestion of live human erythrocytes and that resistant amoebae stain positive with antiserum directed to erythrocyte membrane antigens (35). Though this previous study described ingestion of erythrocytes as erythrophagocytosis, we now know that amoebae are also capable of performing trogocytosis on live erythrocytes (19). In older literature, amoebae were also seen to ingest bites of erythrocytes in a process that was termed microphagocytosis (47). Therefore, we propose a model in which invasive amoebae are able to evade complement detection in the blood by undergoing trogocytosis of human cells and subsequently displaying human cell membrane proteins.

Other mechanisms of complement resistance in E. histolytica, such as mimicry of the complement regulatory protein CD59 (48, 49), an inhibitor of the membrane attack complex (MAC), have been described. Amoebic cysteine proteinases play a role in cleavage of complement components (50–52). Amoebae are also made temporarily resistant to complement lysis through treatment with increasing doses of heat-inactivated human serum, though the mechanism remains unclear (37, 53), and it was recently found that amoebae do not develop resistance to serum from rats by this method (54). As the percentage of amoebae lysed after exposure to human serum in our assays never reached 100%, even under conditions where amoebae were incubated alone, it is likely that multiple factors contribute to complement resistance in E. histolytica, including display of human cell membrane proteins.

It will be of great interest to determine which human proteins are displayed by amoebae. It is possible that complement regulatory proteins, such as CD55 and CD46, are displayed by amoebae and that this directly promotes resistance to complement lysis. Displayed human cell membrane proteins might also bind to soluble factors in human serum, such as factor H. It is notable that acquired human cell membrane proteins do not have an even distribution on the amoeba surface and instead appear in foci. The staining pattern was similar for both biotin-streptavidin and MHC I staining. In mammalian immune cells, similar focal localization of acquired membrane proteins are seen with biotin-streptavidin staining, fluorescently tagged proteins, and immunofluorescence (24, 55). It is not clear if the acquired membrane proteins are present in lipid microdomains (e.g., lipid rafts). It is also possible that while patchy foci of acquired membrane proteins are clearly seen, these proteins might also be found throughout the membrane at concentrations below the limit of detection. In any case, the distribution of human cell proteins appears sufficient to confer protection from complement.

We used *Eh*ROM1 knockdown mutants that were defective in phagocytosis but not defective in trogocytosis to test whether protection was specifically associated with trogocytosis. Consistent with the original description of *Eh*ROM1 mutants (40), we found that they were still capable of attachment to human cells but were defective in attaching to multiple human cells at the same time. Amoebae attached to numerous human cells are termed “rosettes.” The phenotype of *Eh*ROM1 mutants is consistent with the E. histolytica literature in general, where attachment phenotypes do not manifest as abolishment of all attachment but instead result in decreased rosette formation compared to that of control amoebae (8, 40, 56). Since most *Eh*ROM1 mutants still attached to at least one human cell, it appears that the lowered levels of attachment were sufficient to allow trogocytosis to proceed and to allow acquisition of human cell membrane proteins, since the mutants were still protected from lysis by human serum.

With the discoveries of amoebic trogocytosis and display of human cell membrane proteins, a new paradigm for amoeba-host interactions is emerging. We previously showed that when amoebae kill cells, they do not ingest dead cell corpses (19). Prior to this, amoebae were thought to fully ingest the corpses of the cells that they had killed (5, 57, 58). Now, with the discovery of acquisition and display of human cell membrane proteins, together with the lack of ingestion of cell corpses, a different paradigm is emerging. It is possible that rather than acquiring nutrition by killing and ingesting entire cells, amoebae nibble and acquire membrane proteins that contribute to immune evasion. Invasive disease involves survival of amoebae in blood vessels. Since trogocytosis contributes to tissue invasion (19), it is possible that amoebae acquire human cell membrane proteins as they invade the intestine. Amoebae would then be equipped to survive in the bloodstream and to spread to other tissues. Moreover, since there is the potential for a variety of human cell proteins to be displayed, display of human cell proteins may impact amoeba-host interactions in many ways.

Display of human cell proteins acquired during trogocytosis is a novel strategy for immune evasion by a pathogen. Since other microbial eukaryotes use trogocytosis for cell killing, including Naegleria fowleri, there is the potential for display of acquired membrane proteins to apply to the pathogenesis of other infections. Furthermore, our studies extend acquisition and display of membrane proteins beyond mammalian immune cells, suggesting that this may be a fundamental feature of eukaryotic trogocytosis. How membrane proteins are acquired and displayed by immune cells during trogocytosis is not well understood. Thus, ongoing studies of amoebae may shed light on acquisition and display of membrane proteins during trogocytosis in general.

In summary, amoebae display human cell membrane proteins on their surfaces and are protected from lysis by human serum after trogocytosis. We propose a new model of immune evasion by E. histolytica whereby amoebae survive complement attack in the bloodstream through trogocytosis and display of human cell membrane proteins. This work broadens our understanding of trogocytosis as a conserved feature of eukaryotic biology, as well as our understanding of the pathogenesis of amoebiasis.

## MATERIALS AND METHODS

### Cell culture.

HM1:IMSS (ATCC) E. histolytica trophozoites (amoebae) were cultured at 35°C in TYI-S-33 medium supplemented with 80 U/ml penicillin, 80 µg/ml streptomycin (Gibco), 2.3% Diamond vitamin Tween 80 solution (40×; Sigma-Aldrich), and 15% heat-inactivated adult bovine serum (Gemini Bio-Products). Amoebae were harvested when tissue culture flasks reached 80 to 100% confluence and then resuspended in M199s medium (Gibco medium M199 with Earle’s salts, l-glutamine, and 2.2 g/liter sodium bicarbonate, without phenol red) supplemented with 5.7 mM l-cysteine, 25 mM HEPES, and 0.5% bovine serum albumin.

Human Jurkat T cells from the ATCC (clone E6-1) were cultured at 37°C and 5% CO_2_ in RPMI 1640 medium (Gibco; RPMI 1640 with l-glutamine and without phenol red) supplemented with 10 mM HEPES, 100 U/ml penicillin, 100 µg/ml streptomycin, and 10% heat-inactivated fetal bovine serum (Gibco). Human cells were harvested when numbers reached between 5 × 10^5^ and 2 × 10^6^ cells/ml and were resuspended in M199s medium.

### Generation of *Eh*ROM1 mutants.

The *Eh*ROM1 silencing construct, made from a pEhEx plasmid backbone, was generated by Khalil et al. as described previously (59). The construct contained 132 bp of the trigger gene, EHI_048600, fused to 525 bp of *Eh*ROM1 (EHI_197460) (AmoebaDB: https://amoebadb.org/amoeba/app/record/gene/EHI_197460). Amoebae were transfected with 20 µg of the *Eh*ROM1 silencing construct using Attractene transfection reagent (Qiagen). Transfectants were then maintained under selection with Geneticin at 6 µg/ml. Clonal lines were generated by limiting dilution in a 96-well plate contained in a BD GasPak EZ pouch system (BD Biosciences), and silencing was confirmed with reverse transcriptase (RT) PCR. An individual clonal line was used for all experiments. A vector control line was generated by transfection with the pEhEx trigger construct backbone, using the same approach.

### Confocal immunofluorescence assays.

In the biotin transfer experiments, human cells were resuspended in 1× Dulbecco’s phosphate-buffered saline (PBS; Sigma-Aldrich) and then biotinylated with EZ-Link sulfo-NHS-SS-biotin (sulfosuccinimidyl-2-[biotinamido]ethyl-1,3-dithiopropionate) (Thermo Fisher Scientific) at 480 µg/ml in 1× PBS for 25 min at 4°C. One molar Tris-HCl (pH 8) was added to the samples for a final concentration of 100 mM to quench the reaction. Cells were next washed in 1× PBS containing Tris-HCl (pH 8) at 100 mM and then resuspended in M199s. Amoebae were washed and labeled in M199s with CellTracker green 5-chloromethylfluorescein diacetate (CMFDA; Invitrogen) at 310 ng/ml for 10 min at 35°C. Amoebae and human cells were combined at a 1:5 ratio in M199s and coincubated for 5 min at 35°C. Following coincubation, cells were fixed with 4% paraformaldehyde (Electron Microscopy Sciences) for 30 min at room temperature and stained with an Alexa Fluor 633 streptavidin conjugate (Invitrogen) at 20 µg/ml for 1 h at 4°C. After fixation, samples were stained with DAPI (4′,6-diamidino-2-phenylindole; Sigma-Aldrich) for 10 min at room temperature. Samples were then incubated on coverslips precoated with collagen (collagen I, rat tail; Gibco), according to the manufacturer’s instructions, for 1 h at room temperature and mounted on glass slides using Vectashield (Vector Laboratories). In some experiments, samples were incubated on Superfrost Plus microslides (VWR) for 1 h, and coverslips were then mounted with Vectashield. Samples were imaged on an Olympus FV1000 laser point-scanning confocal microscope or on an Intelligent Imaging Innovations hybrid spinning-disk confocal microscope. Images, including 76 images of amoebae with human cells and 21 images of amoebae alone, were collected from 4 independent experiments.

For the MHC class I immunofluorescence experiments, human cells and amoebae were separately washed and resuspended in M199s. Amoebae and human cells were then combined at a 1:5 ratio in M199s and coincubated for 5 min at 35°C. Following coincubation and fixation with 4% paraformaldehyde, samples were blocked for 1 h in PBS-T (0.1% Tween 20 in 1× PBS) supplemented with 20% goat serum (Jackson Immunoresearch Labs Inc.) and 5% bovine serum albumin. Samples were then washed in PBS-T and incubated overnight with an MHC class I monoclonal primary antibody (Thermo Fisher Scientific HLA-ABC monoclonal antibody W6/32) at 10 µg/μl, followed by washing with PBS-T and incubation with an anti-mouse Cy3 secondary antibody (Jackson Immunoresearch Laboratories Inc.) at 3.5 ng/ml at room temperature for 1 h. Samples were stained with DAPI and mounted on glass slides as described above. Images, including 83 images of amoebae with human cells and 40 images of amoebae alone, were collected from 4 independent experiments.

### Imaging flow cytometry immunofluorescence assays.

Amoebae were resuspended in M199s media and pretreated with cytochalasin D (Sigma-Aldrich) at 20 µM or with the equivalent volume of dimethyl sulfoxide (DMSO) for 1 h at 35°C. Cytochalasin D and DMSO were kept in the media for the duration of the experiment. Following pretreatment, amoebae were labeled with CellTracker green CMFDA at 93 ng/ml for 10 min at 35°C. Human cells were labeled in culture with Hoechst 33342 dye (Invitrogen) at 5 µg/ml for 1 h at 37°C and then resuspended in 1× PBS. Human cells were then biotinylated with EZ-Link sulfo-NHS-SS-biotin at 480 µg/ml in 1× PBS for 25 min at 4°C. One hundred millimolar Tris-HCl (pH 8) was used to quench the reaction, cells were washed in 100 mM Tris-HCl (pH 8) and were resuspended in M199s. Amoebae and human cells were combined at a 1:5 ratio in M199s and coincubated for 5 min at 35°C. After coincubation, samples were immediately placed on ice to halt ingestion, stained with an Alexa Fluor 633 streptavidin conjugate at 20 µg/ml for 1 h at 4°C, and fixed with 4% paraformaldehyde for 30 min at room temperature. Fixed samples were resuspended in 1× PBS and run on an Amnis ImageStreamX Mark II. Ten thousand events per sample were collected from six repeats across three independent experiments.

### Serum lysis assays.

Amoebae were washed and labeled in M199s with CellTracker green CMFDA at 93 ng/ml for 10 min at 35°C. Human cells were washed and labeled in M199s with Diic18(5)-Ds (1,1-dioctadecyl-3,3,3,3-tetramethylindodicarbocyanine-5,5-disulfonic acid [DiD]; Assay Biotech) at 21 µg/ml for 5 min at 37°C and 10 min at 4°C. After being washed with M199s, amoebae and human cells were combined at a 1:5 ratio in M199s and coincubated for 1 h at 35°C or amoebae were incubated under the same conditions in the absence of human cells. Next, cells were pelleted at 400 × *g* for 8 min and were resuspended in 100% normal human serum (pooled normal human complement serum; Innovative Research Inc.), heat-inactivated human serum (inactivated at 56°C for 30 min), or M199s. Serum/medium was supplemented with 150 µM CaCl_2_ and 150 µM MgCl_2_ (see Fig. S2 in the supplemental material). Next, cells were incubated for 30 min at 35°C. Cells were then washed and resuspended in M199s media and incubated with Live/Dead fixable violet dead cell stain (Invitrogen) that was prepared according to the manufacturer’s instructions at 4 µl/ml for 30 min on ice. Next, samples were fixed with 4% paraformaldehyde for 30 min at room temperature. Fixed samples were pelleted and resuspended in 1× PBS and then run on an Amnis ImageStreamX Mark II. Ten thousand events per sample were collected.

In the cytochalasin D experiments, amoebae were pretreated with cytochalasin D at 20 µM or with an equivalent volume of DMSO for 1 h at 35°C. Cytochalasin D/DMSO was kept in the media for the duration of the experiment. In experiments where amoebae ingested live or prekilled cells, human cells were pretreated in culture with staurosporine (Sigma-Aldrich) at 1 µM or with the equivalent volume of DMSO overnight at 37°C. Human cells were then washed and suspended in M199s media and labeled with CellTracker deep red (CTDR) (Invitrogen) at 1 µM for 30 min at 37°C. In transwell assays, amoebae and human cells were incubated together at a 1:5 ratio or separately in 12-mm transwells with 3.0-μm-pore-size, 10-μm-thick polycarbonate membrane inserts (Corning). In experiments using EhROM1 knockdown, stably transfected *Eh*ROM1 clonal mutants were compared to mutants that contained a pEhEx trigger backbone vector control construct.

### Ingestion assays.

In trogocytosis assays, CMFDA-labeled transfectants were incubated alone or in the presence of live DiD-labeled Jurkat cells for 0, 5, 20, 40, or 80 min. Samples were then labeled with Live/Dead violet and fixed with 4% paraformaldehyde. Internalization of human cell material was quantified using imaging flow cytometry. In phagocytosis assays, human cells were heat killed at 60°C for 40 min and were labeled with CTDR and Hoechst dyes prior to incubation with CMFDA-labeled amoebae.

### Attachment assay.

CMFDA-labeled amoebae were combined with CTDR-labeled live human cells at a 1:5 ratio, centrifuged at 150 × *g* for 5 min 4°C, and incubated on ice for 1 h. Samples were then fixed with 4% paraformaldehyde. Samples were incubated on Superfrost Plus microslides (VWR) for 1 h, coverslips were mounted with Vectashield, and slides were imaged on an Intelligent Imaging Innovations hybrid spinning-disk confocal microscope. Twenty images were collected per slide. Amoebae with 3 or more attached human cells were scored as attachment positive. Image collection and scoring were performed in a blind manner.

### Imaging flow cytometry analysis.

Samples were run on an Amnis ImageStreamX Mark II, and 10,000 events were collected per sample. Data were analyzed using Amnis IDEAS software. Samples were gated on focused cells, single amoebae, amoebae that had come in contact with human cells, and amoebae that had internalized human material. From the single amoeba gate, amoebic death was quantified by plotting the intensity of Live/Dead violet against side scatter and gating on Live/Dead violet-positive cells (Fig. S3).

In the biotin transfer experiment, single amoebae were divided into high-Hoechst and low-Hoechst populations in order to isolate single amoebae with and without human cells. Overlap of biotin with CMFDA-labeled amoebae was plotted, and biotin-positive cells were selected from both high-Hoechst and low-Hoechst populations (Fig. S1).

For calculation of the background level of phagocytosis, the imaging flow cytometry data from the experiments shown in Fig. 2 were used. The DMSO control-treated amoebae were used for this analysis. Single amoebae were gated from total cells. Next, biotin-positive amoebae were gated. Amoebae associated with human cell nuclei that were surrounded by a biotin-streptavidin ring were considered phagocytosis negative, while amoebae associated with human cell nuclei that lacked a biotin ring were considered phagocytosis positive. Amoebae that were not associated with human cell nuclei were considered phagocytosis negative. Some amoebae were out of focus or were associated with too many human cells to reliably score; thus, these images were left unscored. The first 100 images in the biotin-positive amoeba gate that could be scored (300 total images from three independent experiments) were analyzed. Since some images were unscored, more than 100 total images were analyzed per experiment, as indicated in the raw-data table. Images were counted independently by two different researchers, and the counts were averaged.

In the trogocytosis and phagocytosis assays, focused cells were gated from total collected events. Next, single cells were gated, and then single amoebae were gated. Amoebae positive for human cells were gated, and internalization of human cells was measured (Fig. S5).

### Statistical analysis.

All statistical analyses were performed using GraphPad Prism. All data plots display means and standard deviation values. Data were statistically analyzed using Student’s unpaired *t* test (no significant difference was indicated by a *P* of >0.05; *, *P* ≤ 0.05; **, *P* ≤ 0.01; ***, *P* ≤ 0.001; ****, *P* ≤ 0.0001).
