# Supplementary figures and images for: Trogocytosis by Entamoeba histolytica Mediates Acquisition and Display of Human Cell Membrane Proteins and Evasion of Lysis by Human Serum
Source: mBio. 2019 Apr 30;10(2):e00068-19. doi: 10.1128/mBio.00068-19 (PMC6495370; doi:10.1128/mBio.00068-19)

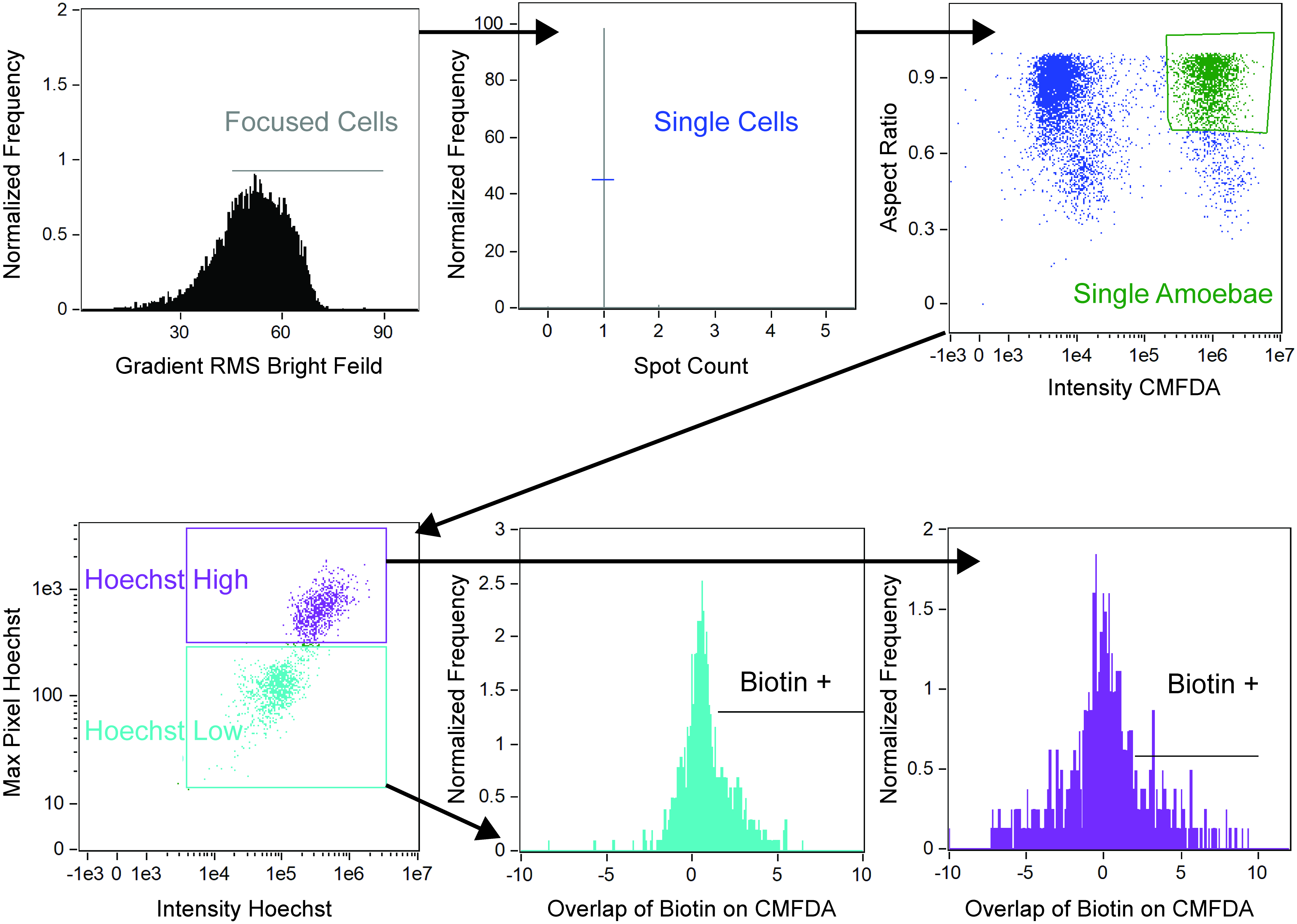

Supplement: FIG S1 [file mBio.00068-19-sf001.tif]

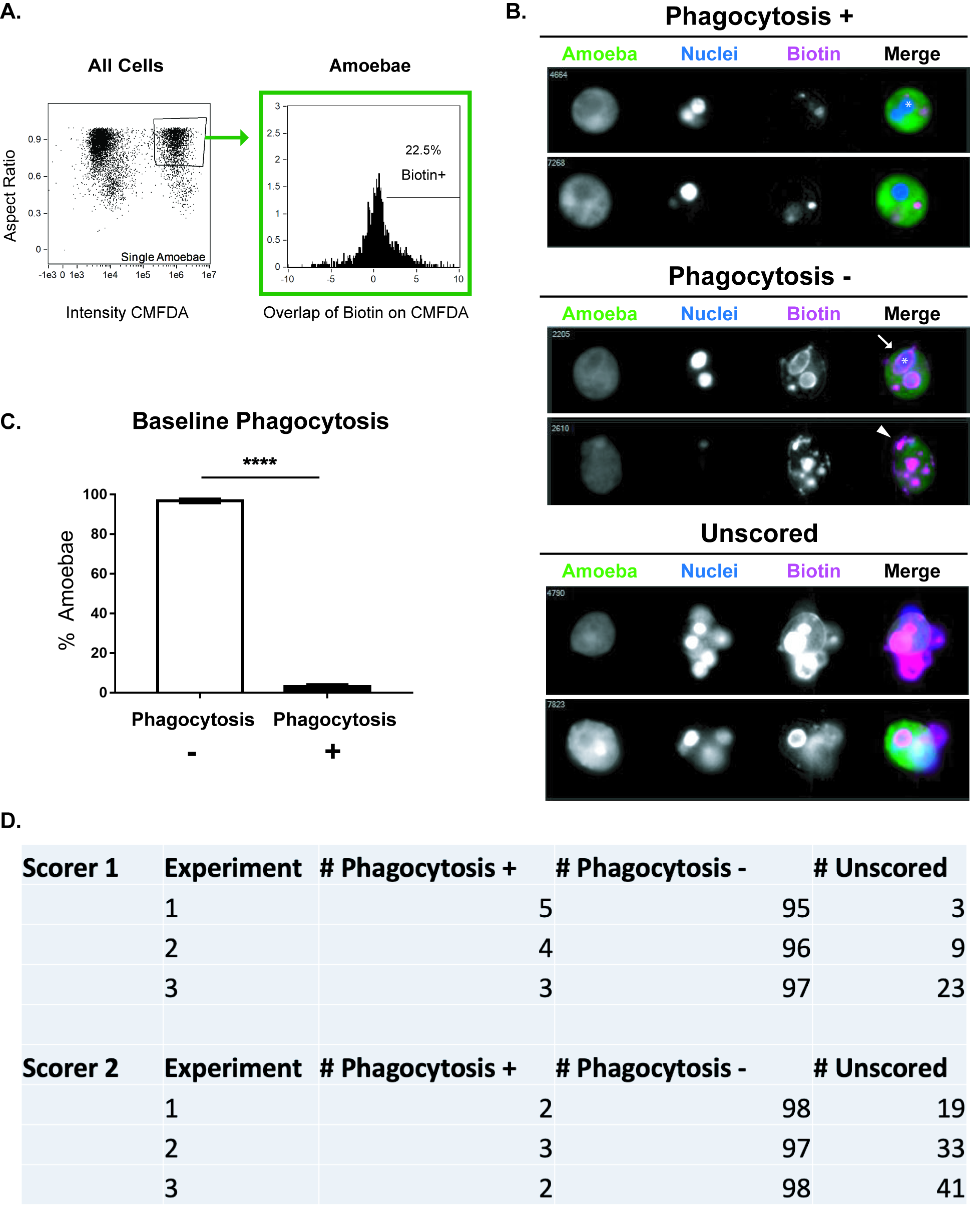

Supplement: FIG S2 [file mBio.00068-19-sf002.tif]

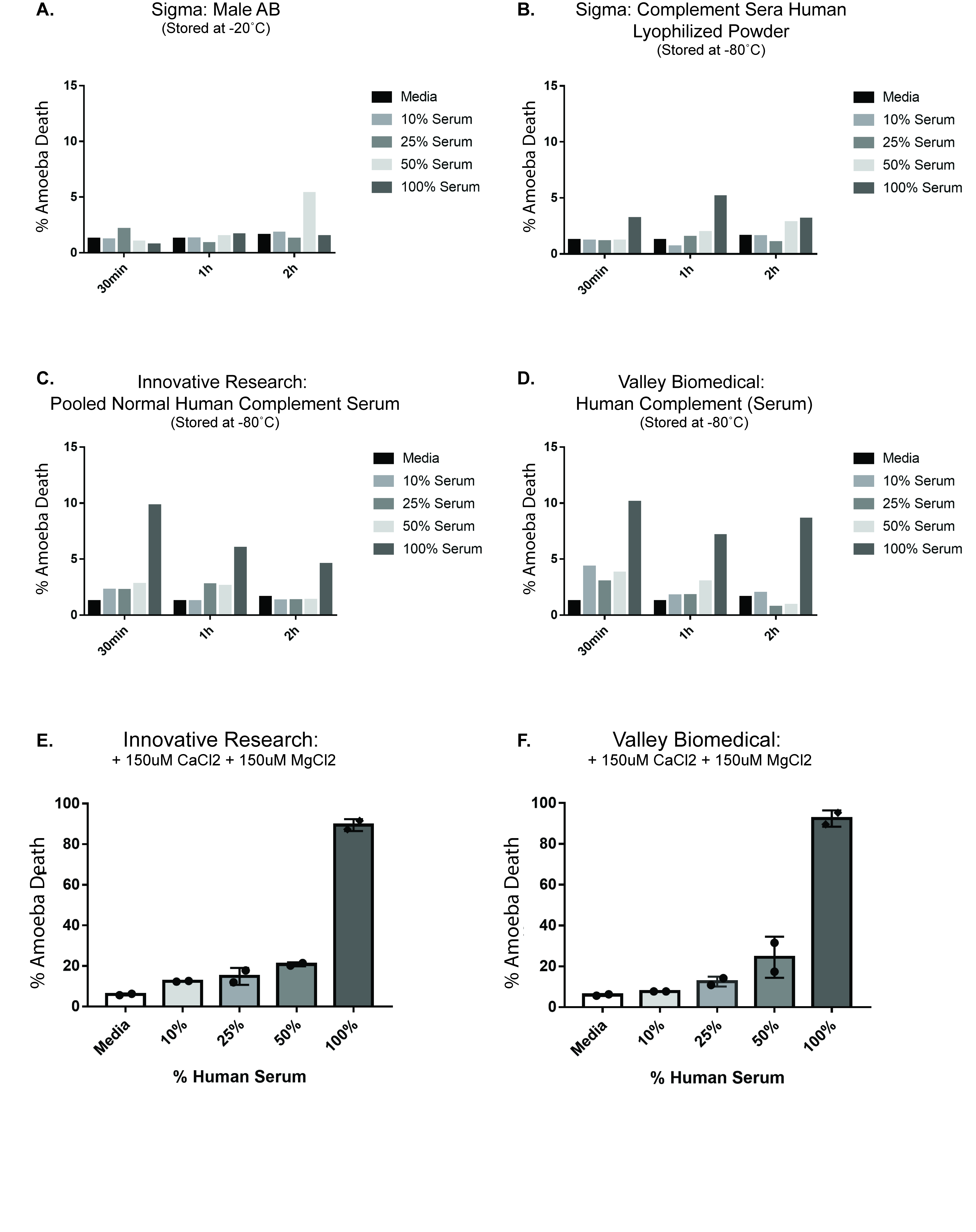

Supplement: FIG S3 [file mBio.00068-19-sf003.tif]

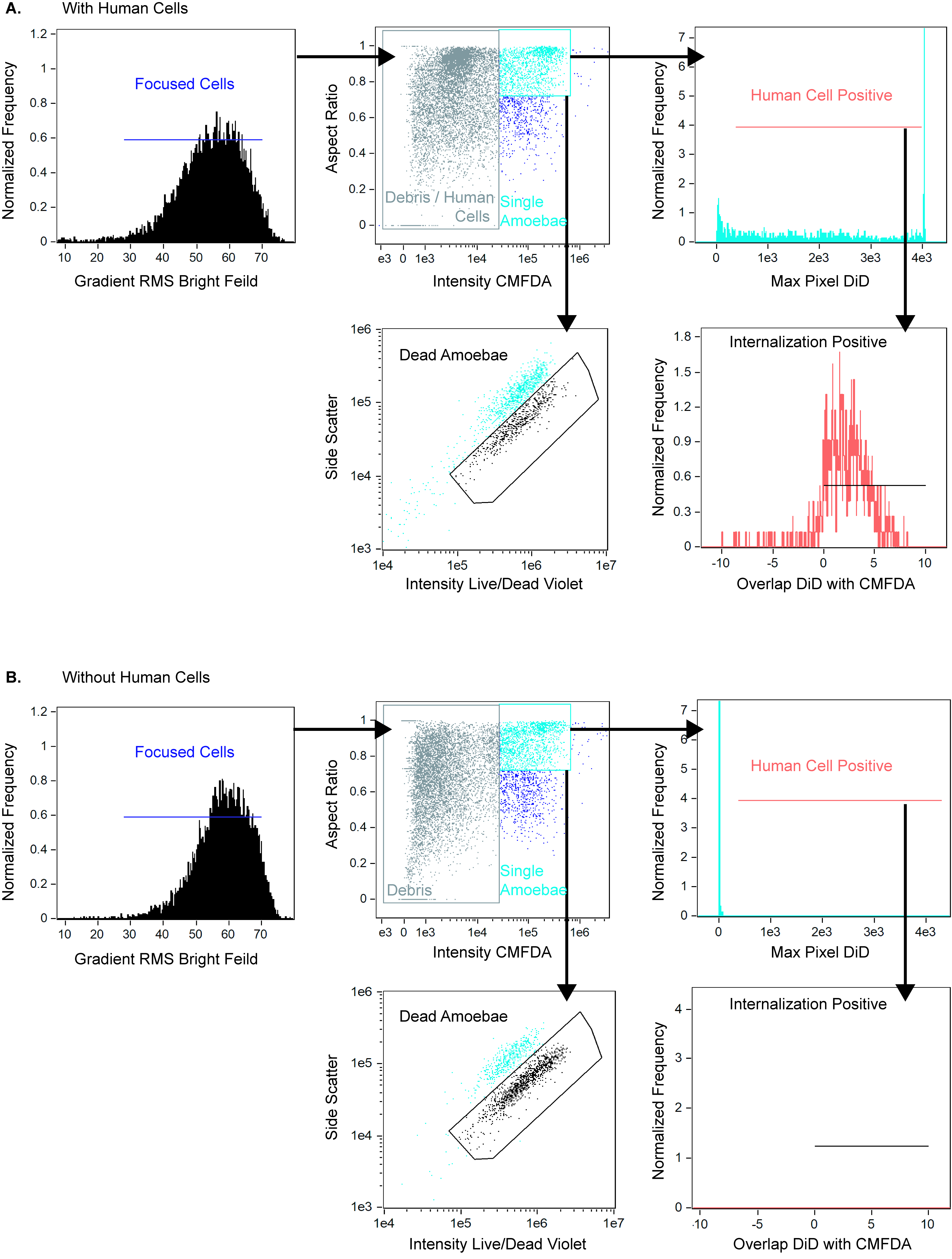

Supplement: FIG S4 [file mBio.00068-19-sf004.tif]

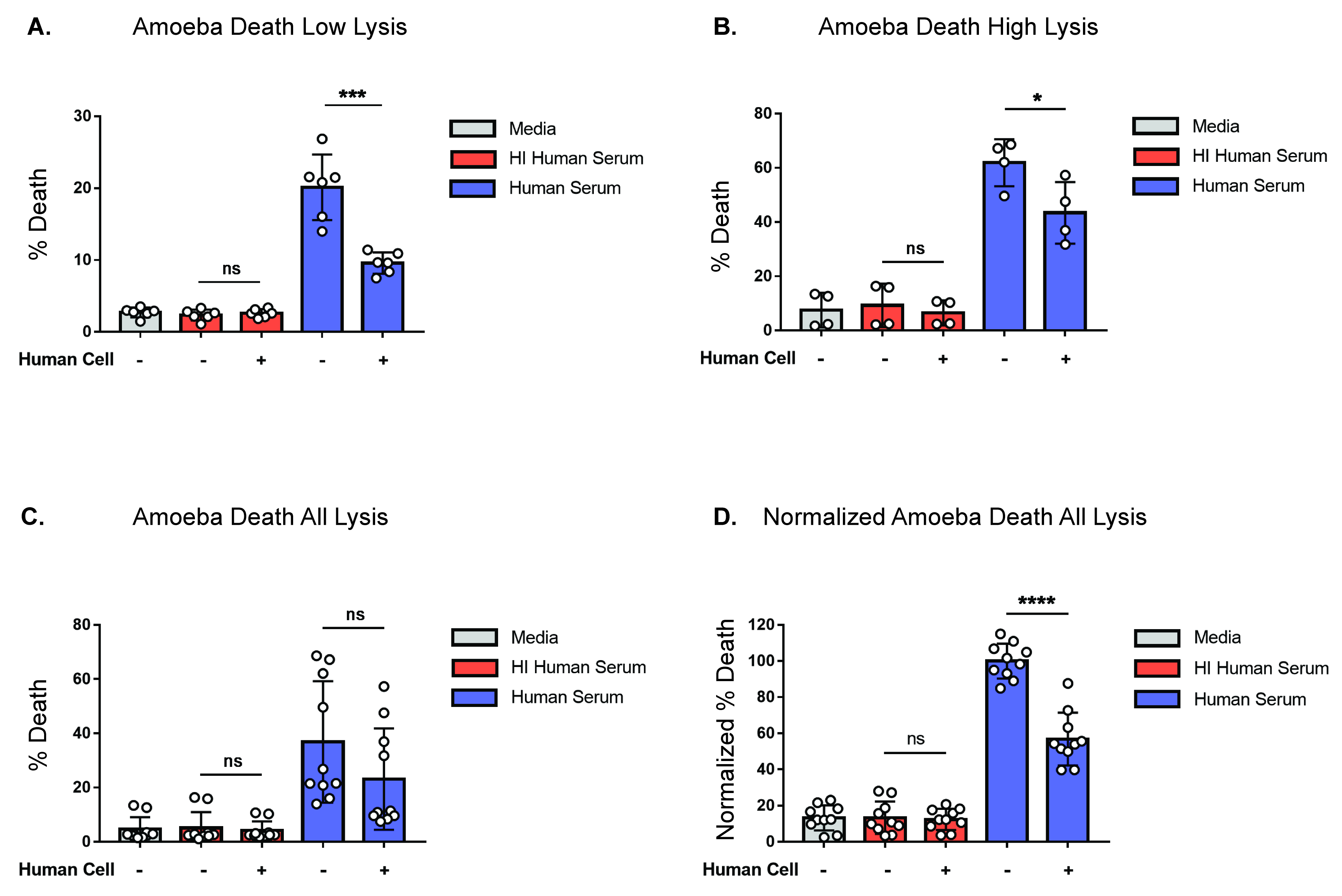

Supplement: FIG S5 [file mBio.00068-19-sf005.tif]

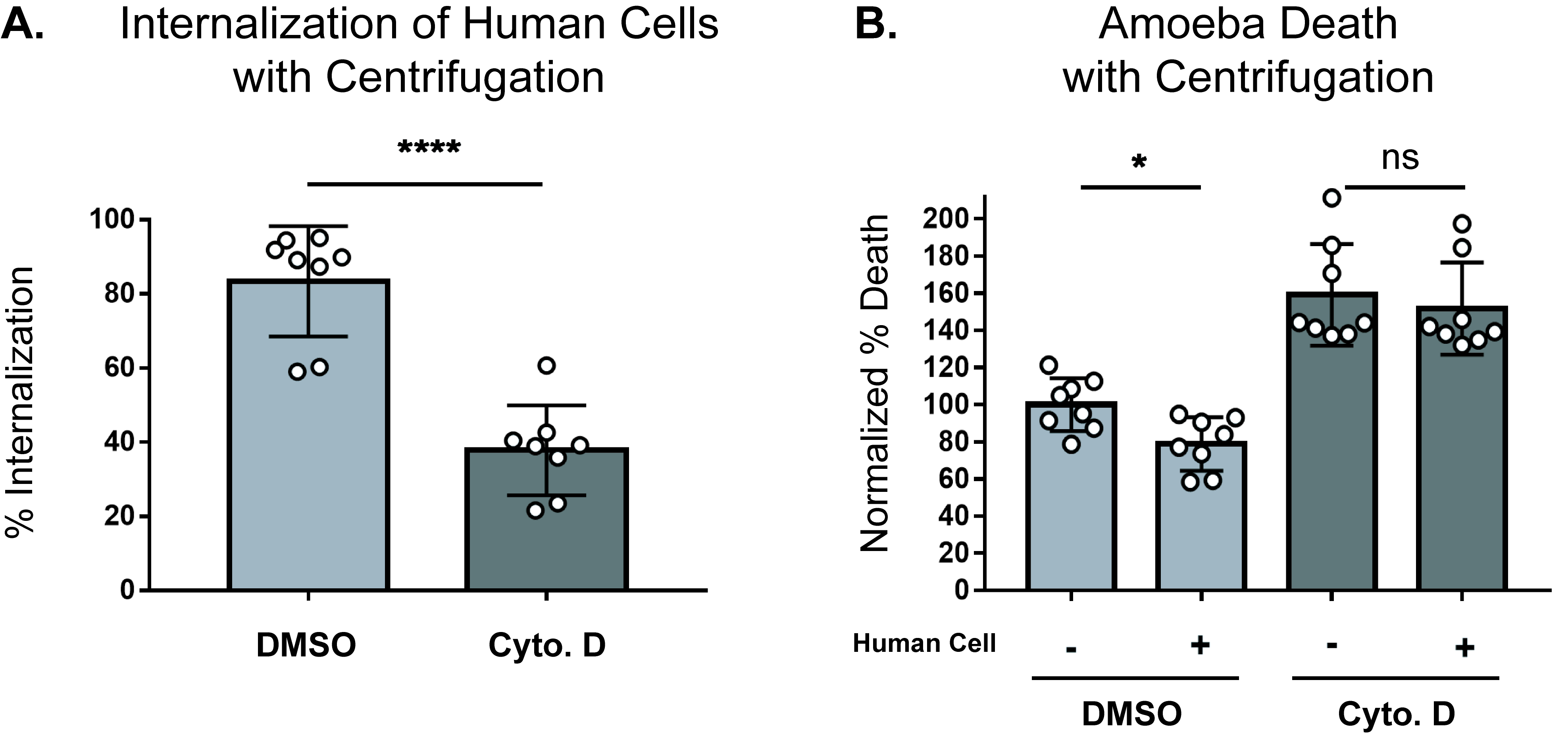

Supplement: FIG S6 [file mBio.00068-19-sf006.tif]

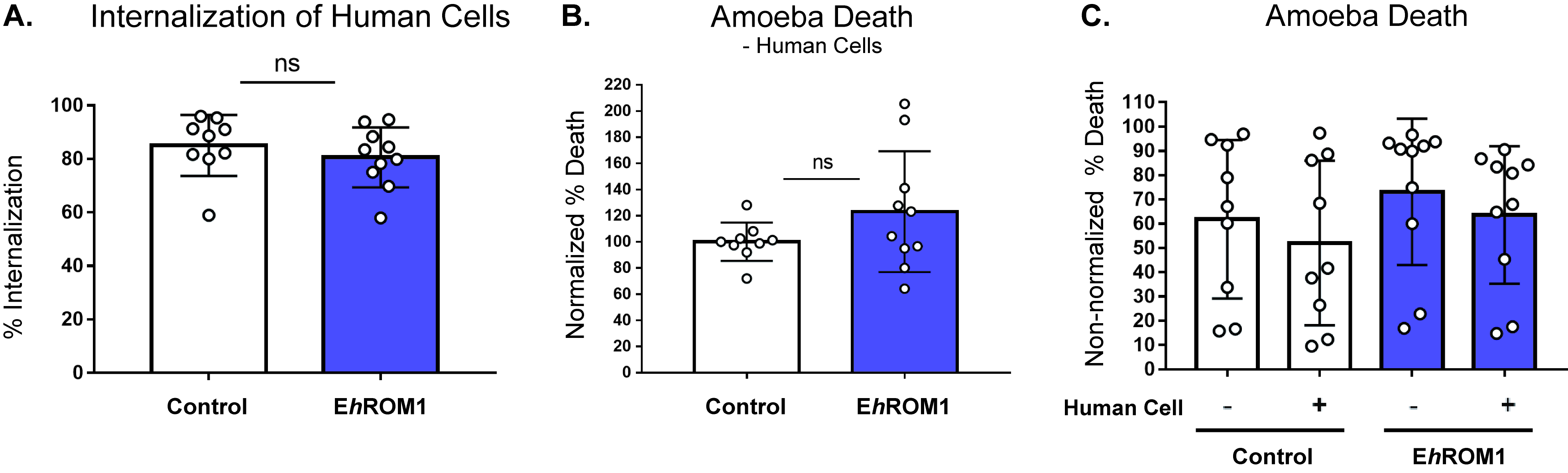

Supplement: FIG S7 [file mBio.00068-19-sf007.tif]

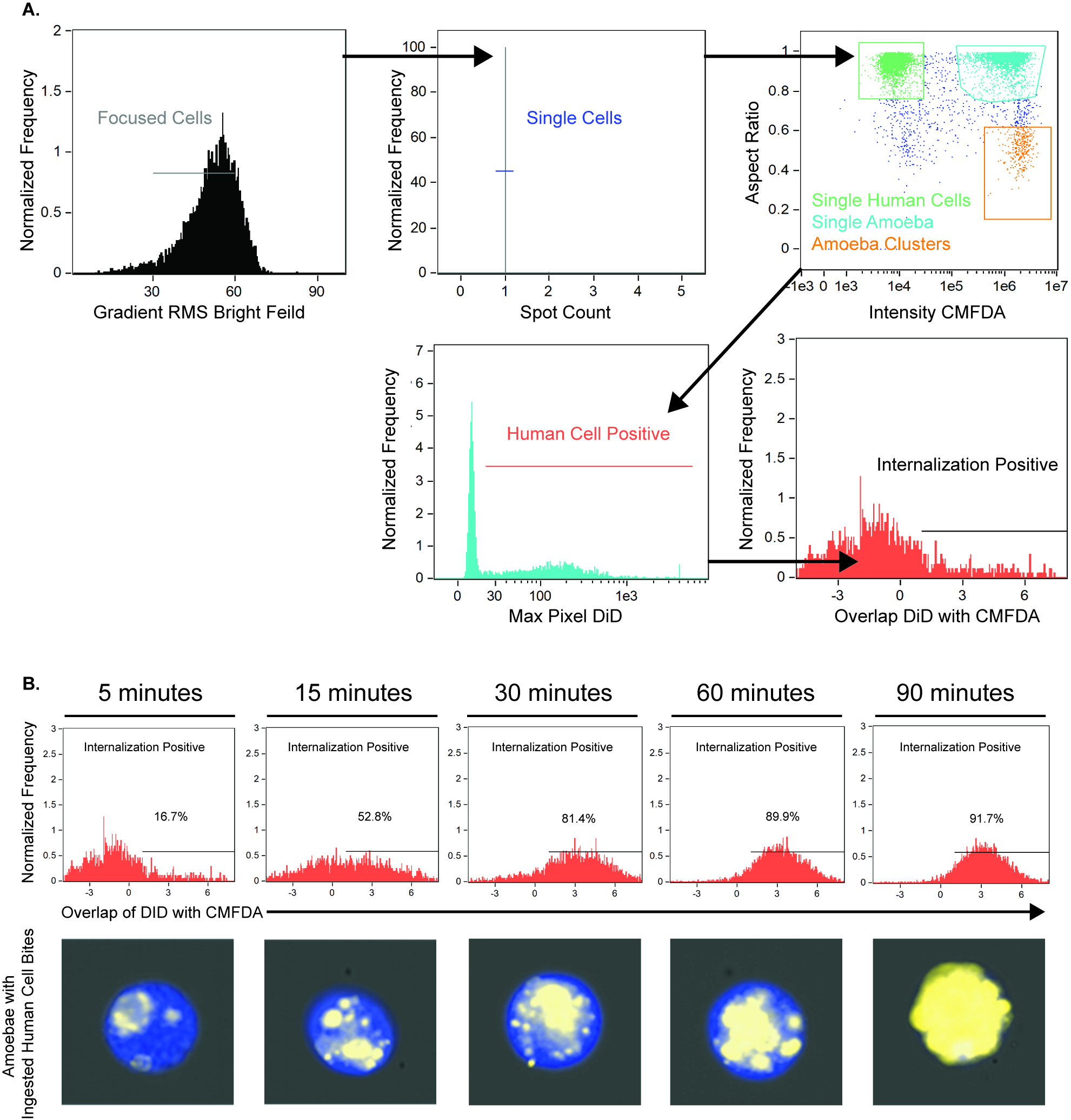

Supplement: FIG S8 [file mBio.00068-19-sf008.tif]
